# Hospital and Institutional News

**Published:** 1917-12-29

**Authors:** 


### HOSPITAL AND INSTITUTIONAL NEWS.

### A BOMBARDMENT ANNIVERSARY.

Sunday, December 16, was the anniversary of the bombardment of the Hartlepools, and it is characteristic of the unconscious humour of the Englishman to celebrate the anniversary of the occasion. A German would celebrate the anniversary of an outrage upon other people—the *Lusitania* medal was a symptom of his mood. An Englishman prefers to celebrate the occasion of an outrage upon himself. He has made it the occasion of a "Thankoffering Organisation," devoted to the assistance of the Hartlepool and Cameron Hospitals. "Numerous undertakings," we read, were "in hand in connection with the event." There is a quaint humour in this which cannot be lost upon the keenest sympathiser. So flags were sold and an organ recital was held and a dramatic entertainment. All this is very human and English, and we hope that the hospitals benefited in proportion. That this sort of thing would probably be incomprehensible to the Germans illustrates the contention of those like Mr. Hilaire Belloc that Prussia is outside the pale, or, in other words, beyond the boundary of Europe and the civilisation of the West. We make him a present of the illustration of a fact which has too few merely humorous illustrations.

### WORKERS' FUNDS IN COUNTRY TOWNS.

Hospitals in towns composed of middle-class people and with but a small industrial and trade union element are finding it more difficult to maintain their finances than those in factory centres. A leading case is that of the Royal Sussex County Hospital, Brighton. Here the annual cost per bed has risen from £73 to £93 a year. In 1916 there was a deficit of £2,700. To-day this debt has increased to £4,000, and in 1918 an additional sum of £6,000 will be required by the estimate of the committee, if the policy of accepting soldiers without unduly restricting the admission of civilians is to be continued. The hospital has ninety years of work behind it, and is proud to possess a pathological institute. Its subscribers have been thinned by death, and it is increasingly difficult to maintain their numbers. Therefore the present appeal for £10,000 has been made. While wishing it success, we believe that the committee must recast its financial policy by the sectional organisation of subscriptions. To do this the shopowners of Brighton must be approached and an attempt be made to organise shop collections. Something of the kind has been done at Cambridge, and it is found possible to organise shop collections on the same lines as those in other firms or "works." Without some such attempt even the success of this appeal is not likely permanently to improve the position.

### THE LIMITS OF COMMERCIAL ADVERTISING.

A curious plan has lately been adopted at Reading, where Messrs. Salmon, Ltd., wholesale packet tea dealers, have offered twenty-one guineas to "the advertising man" who submits the most practical scheme for assisting the Royal Berkshire Hospital. A sequel to the scheme, which produced no immediate result, was an offer from Mr. E. S. Daniells, of the Ingersoll Company, of ten guineas for any satisfactory scheme, other than that selected by Messrs. Salmon, for helping the Great Northern Central Hospital. So barren were the firstfruits of this notion that women were deemed to be included in the term "advertising men." We have not yet heard of any startling result for the benefit of either institution. Does either hospital hope much from this curious suggestion? It seems to us silly and undignified for a great institution to countenance an appeal to any section of laymen as to how it shall best assist its finances. The appeal to advertisers can only produce a crop of fantastic suggestions. For the truth is that a hospital's position does not depend upon any series of "appeals," however startling, but on interesting the public in the progress of its work. The work must be presented properly certainly, but the catchpenny method can never be so fruitful as the ideas of those who know and understand the voluntary sytem from within.

### THE "DUMPING" OF PHTHISICAL CASES.

The first meeting has now been held of the Tuberculosis Aid and After-Care Association for "the county borough" of Bournemouth. Practically all the public bodies of the district are represented. The scheme originated with the Insurance Committee, and has received the benediction of the medical officer of health. The aims of the Association are to visit patients, to gain the support of employers, to seek suitable occupation for discharged patients unable to obtain it, and to co-operate with existing bodies in providing adequate nourishment and clothing. The Mayor of Bournemouth, in opening the meeting, declared amid applause that they did not want to set up in the town an establishment which would tend to bring consumptives. A councillor thereupon urged that definite steps should be taken to prevent doctors from other parts "dumping" patients into Bournemouth. We fear that this praiseworthy departure is not likely to be so extensively successful as to cause Bournemouth to become a resort in this sense. If it will, its methods are sure to be copied in other centres.

### ROYAL ALBERT EDWARD INFIRMARY, WIGAN.

Mr. Will Taberner, who recently tendered his resignation as general superintendent and secretary of this institution, has occupied that office for a period of over forty years, and has been associated with the progress and expansion of the infirmary nearly since its inception. During Mr. Taberner's tenure of office many additions have been made to the original building—the Gidlow Wing, the Johnson Wing, the mortuary, including the *post-mortem* and pathological rooms, the nurses' dining-hall, the Gidlow Wing extension, the isolation block, the children's balconies, and the nurses' home. The weekly contribution movement during those years has become progressively more and more successful, and now the magnificent sum of over £8,000 is contributed annually by the workpeople of the Wigan district, a record of £9,308 10s. 1d. being attained in the year before the commencement of the war. Mr. Taberner had ever the welfare and progress of the institution at heart, and the board of management have conveyed to Mr. Taberner upon his resignation their warmest thanks for the services he has rendered to the institution during the many years he has been general superintendent and secretary.

### A SUBSTITUTE FOR CHRISTMAS PLUM-PUDDING

The shortage of fruit, and particularly of currants, has done more than the Food Controller to diminish the consumption of plum-pudding. It is impossible to suggest a satisfactory substitute. But it is interesting to record the alternative approved by a Sussex Board of Guardians. On the master's suggestion ginger pudding is being served to the inmates of his institution. The devotees of Christmas plum-pudding will probably complain that "there is no ginger" in this pudding on Christmas Day.

### A SOLDIER'S LEGACY.

The Rev. Joseph Baker, Vicar of St. Catharine's, Cardiff, has written the following letter to Mr. Leonard Rea, secretary of King Edward VII.'s Hospital, Cardiff: "On July 31 last the secretary of our Sunday schools was killed in Flanders. His mother tells me that she has just heard from the War Office that his will has been found. He was only a clerk, and had but very little of this world's goods, but he has left three bequests, and these bequests, of course, imply that at such a time certain causes were dear to him. (1) King Edward VII.'s Hospital, (2) Y.M.C.A., and (3) the Assistant Clergy Fund of this parish." The parents of the testator have handed over the bequests with the inscription "From Fred, of the 13th Welsh."

### THE ARMY COUNCIL AND FOOD ECONOMY.

The increased capitation grant which has been notified to V.A.D. auxiliary hospitals (3s. to 3s. 3d. for Class "A" hospitals and 2s. to 2s. 6d. for Class "B" hospitals) has been made conditional upon information being accorded to the Army Council of the exact amount of food consumed in the institutions. In consequence, the Central Joint V.A.D. Committee has instructed all auxiliary hospitals to keep "a careful return of all provisions consumed during each month." To enable this to be done special forms are being issued, and the Commandants are asked "as a preliminary, to take stock of all provisions in hand on December 31." These steps are to be commended, but there would appear to be some justification for the Army Council's stipulation, since in every economically managed hospital the monthly stocktaking of provisions is a *sine quâ non*. The efforts of the Army Council to inculcate economy will no doubt be watched with especial interest and sympathy by King Edward's Hospital Fund.

### SANATORIUM TREATMENT.

Representatives of eighteen Metropolitan Borough Councils are to attend a Conference called by the Lewisham Borough Council to consider the present situation of sanatorium treatment. As is well known, the Insurance scheme has broken down, and the Conference is called to discuss what can be done for the consumptives of London. Westminster Borough Council has definitely decided not to take part in the Conference, as it considers that no useful purpose would thereby be served at the present time. The City Corporation is sending delegates, and it is hoped that it may provide accommodation for the Conference in the Guildhall.

### FOR EXTRA SERVICES RENDERED.

Southwark Board of Guardians have unanimously decided to grant Dr. J. F. Williams, medical officer at their Newington institution, an extra allowance of fifty guineas a year from November 1, 1915, and to continue it during the military occupation of their infirmary. When the latter event took place a large number of patients, who could not be accommodated at the Lambeth Infirmary, comprising principally chronic cases and old folks requiring constant medical treatment, were removed to the Newington institution, and these involved a vast amount of additional work and responsibility. This recognition of the services rendered by Dr. Williams is only what naturally would be expected in the exceptional circumstances.

### SURGICAL TUBERCULOSIS AT ALTON.

The good work that has been done and is being done at the Treloar Cripples' Hospital at Alton is known to all, and though the foundation of the hospital is due to the zeal and energy of the ex-Lord Mayor after whom the hospital is named, the great success which has been attained there is due in no small degree to the knowledge and skill of the medical superintendent, Mr. Henry J. Gauvain, and therefore no little interest attached to a lecture which he delivered on December 12 at the Royal Institute of Public Health. He showed that not only are the patients there placed in ideal climatic conditions and have abundant access to fresh air and sunlight; they have abundance of good food, and deformity is prevented and corrected where possible, but in addition all children above the age of five years receive education designed especially to meet their requirements and administered by trained, certificated, and specially selected teachers. Boys who remain crippled when the treatment is completed are drafted where possible to the "College," where they are kept for three years, their education is continued, and they are taught a trade. The results are surprisingly good as a whole. Patients after discharge are kept under observation, they receive both medical and sociological advice, and are directed into such occupations as will afford them the best prospects of remunerative employment, despite any physical disabilities which may persist. Since the hospital was opened 1,890 cases have been admitted, the mortality has been only

1.67 per cent., and 1,517 cases have been discharged with the disease arrested. The hospital and college are doing very excellent work, and they deserve even more support than they receive.

### MINISTRY OF HEALTH.

The Council of the Royal Sanitary Institute, at a special meeting called to consider the various proposals made with regard to the formation of a Ministry of Health, resolved: "That a scheme to establish a Ministry of Health, which must affect so many conflicting interests and concern so many different authorities, both central and local, demands fuller consideration than it has yet received, and the Council of the Royal Sanitary Institute will gladly co-operate with His Majesty's Government in the preparation of any legislation having for its object the improvement and consolidation of the Public Health administration of the country." The subject is not new, for in the middle of last century a "General Board of Health" was in active operation as one of the Government Departments, and its history and functions are fully described in Sir John Simon's "English Sanitary Institutions." After ten years of service, 1848-58, this Board, owing to difficulties that arose, was abolished, and the functions it exercised were distributed. In less than twenty-five years the idea of recombining the administration of Public Health work was again brought forward, and so long ago as 1883 the Sanitary Institute had under consideration proposals for collecting the work under one Government Department. The subject has also received further consideration at several meetings held by the Institute in different parts of the country in later years.

### A STATE MEDICAL SERVICE.

A State Medical Service has no terrors for Dr. T. Evans, the M.O.H. of Swansea, who believes that it will give the profession a new outlook upon disease. At the present time, he points out, medical men are not, as a rule, called in to see a patient until he or she is ill, whereas, a great deal of disease being preventable, it would be the business of a Ministry of Health to see that the people were maintained in a state of health rather than to cure a diseased community. "The people will be sought and the illness prevented," says Dr. Evans. "The child will be discovered in the infants' clinic; the mother will be discovered by visits to the home; the girls will be discovered in the same way as it now done by the factory surgeon, and that will be continued in the continuation schools; and the workman will be discovered in the works."

### A DRASTIC HEALTH BY-LAW.

There is no hesitation over health matters with some of the South African municipalities. In view of a serious outbreak of typhus fever which has occurred in other parts of the Union, the Johannesburg Town Council is asking the Provincial Government to give its sanction to the following by-law: If any person, not being himself sick, has been exposed, or is suspected of having been exposed, to the infection of smallpox, plague, cholera, typhus fever, or any other formidable infectious or pestilential disease, and is so lodged that, in the opinion of the Medical Officer of Health, he either is not effectively isolated or cannot conveniently be kept under observation, the Council may, on a certificate to such effect signed by the Medical Officer of Health, by special order direct the removal of such person to and his detention in a proper place of observation for a period not exceeding the known maximum period of incubation of the disease in question. This is as comprehensive as anyone could wish, and gives the authorities drastic powers to protect the community. In the case of plague and smallpox the by-law has been in operation for some time.

### DEATH IN THE SHAVING-BRUSH.

The shaving-brush has always been suspect when cases of anthrax have occurred, but now steps are being taken to reassure those who shave that there will no longer be risk of an early grave through infection. At the request of the Medical Inspector of Factories, the Walsall Corporation has arranged to disinfect horse-hair required for the local manufacture of shaving-brushes, it being understood that whilst every care will be taken to avoid doing any damage to the horse-hair, the Corporation cannot accept any responsibility in this respect. This is progress, and the marvel is that arrangements for such disinfection were not made years and years ago.

### BRITISH RED CROSS IN HOLLAND.

The activities of the Red Cross are ubiquitous, and the latest centre of their work is Holland, where an influential committee has recently been formed under the presidency of Lady Susan Townley, the wife of the British Minister. Already 13,000 gulden (about £1,300) has been subscribed without appealing to the central organisation in London. The Baroness van Brienen has placed her mansion at Klingendaal at their disposal as a hospital for officers suffering from shell-shock, neuroses of various kinds, barbed-wire disease, etc. The Dutch Government has consented to allow the Red Cross authorities to undertake the training and education of prisoners of war and interned civilians, and efforts are being made to get into touch with professors capable of giving lessons and lectures in foreign languages. A similar work has already been organised on a large scale in Switzerland, and there is no doubt that when arrangements have been completed a most valuable equipment will have been given to the men preparatory to their return to civilian life.

### HOSPITAL "CINEMAS" AND ARMY IDIOMS.

It is useless to withstand the popular word for a new thing which, with the main virtue of brevity, has usually no other claim to respect. Thus we all accept telegram, instead of telegraphema, cinema for cinematograph, taxi for taximeter, cab for cabriolet, and the like. The still briefer and purely idiomatic names are, however, much better, on the principle that extremes meet at their central point. Thus "wire" has virtually replaced the monstrosity "telegram," except on the official form. And for cinema with a wrongly short "e," "the movies" is much more racy and intelligible. The line where idiom ends and slang begins is a nice one, but hospital men with a tincture of science and medical nomenclature have usually a more or less lively interest in observing it. We are prompted to these reflections by seeing in print an announcement that a cinematograph theatre has been installed in Bournbrook Hospital, Birmingham, which vies with some London hospitals in being early in this field. What do the Tommies call it? Is the term "movies," so popular with London children, used by the men in blue, or have they an invented army name like the adjective "umpteen" which is commonly applied by the men to a variety of otherwise innocent objects.

### FOOD AND HEALTH.

During the dock strike a few years ago labour leaders sought to invoke public sympathy by statements that children in East London districts were dying of starvation. As the strike had been on some time, it seemed obvious that there must be acute distress, but, remarkably enough, the health returns issued shortly afterwards by the Stepney Medical Officer of Health revealed that during the strike period the death-rate amongst children was lower than usual, and that children had not been dying of starvation, notwithstanding the strike. This incident is recalled by reason of a report just to hand from the Burgh of Peterhead, Scotland, It seems that a headmaster of one of the schools declared that many children were suffering from diarrhœa, doubtless as the result of improper feeding, and he pointed out that of 433 children 70 had only a small bowlful of milk, 273 only a cupful or less, and 90 had no milk at all. Dr. Gillespie, the Medical Officer of Health, has made investigations, and reports that though the headmaster may be quite correct in regard to improper feeding, the suggestion that the illness was partly caused by a shortage of the milk supply could not be substantiated. Inquiries made amongst milkmen by Dr. Gillespie prove that there was no scarcity, and he expresses the opinion that the fact that certain children received none was due either to the price being too high or certain parents not caring to put themselves to the trouble of providing their children with porridge and milk, but preferring to give them bread and tea instead.

### A "KHAKI HANDICRAFTS CLUB" AT BRADFORD.

A meeting was held in Bradford recently to found an industrial club for the shell-shock cases which are being treated at a special hospital in the town. The importance of graduated employment is one that has been widely recognised of late in the treatment of this class of case, but an innovation is being introduced at Bradford in keeping the work centre entirely separate from the hospital and making it a recreation place as well as a workshop. The men are to be taught trades involving finely graded movements, such as mat-making, wood carving, and netting, since it is believed that these are the most effective in aiding the patient to regain his natural powers. The directors of the Midland Railway Company have, we understand, granted free use of certain premises in a central position, and, if the commercial reputation of Bradford is to be relied on, it will not be long before the scheme is working upon a satisfactory basis.

### THE WAR-PRICE OF DRUGS.

In 1917 the rate of the advance in the prices of drugs has been much less violent than it was during the first two years of the war. In fact, the end of the year finds several commonly prescribed drugs at a lower value than did the beginning; but these are exceptional cases since the upward movement of prices has in the majority of instances continued throughout the year, and seems likely to persist until the end of the war. When one considers how large a proportion of our materia medica is derived from oversea sources this is not surprising. The remarkable thing is that the number of drugs that hospitals find a difficulty in obtaining is so very small; but the longer the day of victory is delayed the longer will become the list. Is there any drug so indispensable as to be more important than shells and men? If there be such a drug, we may depend upon it that the ends of the earth will be searched for supplies. When we speak as civilians of the scarcity of drugs we do not mean that the Medical Service of our armies is under-supplied; the needs of this Service are, and must be, satisfied first, and what is left over is available for distribution, through the ordinary commercial channels, among civilian hospitals and the ordinary population. When, therefore, one hears that such-and-such a drug is difficult to obtain, it does not necessarily follow that it is being produced in less quantities than in normal times. On the contrary, the vast numbers of men that have been wounded, or fallen victims to one or other of the diseases to which fighting men in the many theatres of the war—with their different climates and conditions of life—are liable, have necessitated the finding somehow or other of vast quantities of drugs. Sometimes this necessity has been met by larger production, or by ransacking the store-houses of the world; and sometimes by reducing the quantities available to the civil population. The same thing applies to all commodities. It applies, for instance, to foodstuffs; the Army must come first. In the case of drugs we have heard of few hardships. We have heard of no cod-liver oil queues, for instance; nor are we likely to hear of them. What the war has taught us, however, is that a drug that is scarce can be replaced, often without disadvantage, by one that is plentiful; and the longer the war lasts the more thorough will become this teaching.

